# Skeletal stem cells in bone development, homeostasis, and disease

**DOI:** 10.1093/procel/pwae008

**Published:** 2024-04-06

**Authors:** Guixin Yuan, Xixi Lin, Ying Liu, Matthew B Greenblatt, Ren Xu

**Affiliations:** The First Affiliated Hospital of Xiamen University-ICMRS Collaborating Center for Skeletal Stem Cell, State Key Laboratory of Cellular Stress Biology, School of Medicine, Faculty of Medicine and Life Sciences, Xiamen University, Xiamen 361005, China; Xiamen Key Laboratory of Regeneration Medicine, Fujian Provincial Key Laboratory of Organ and Tissue Regeneration, Organ Transplantation Institute, School of Medicine, Xiamen University, Xiamen 361102, China; The First Affiliated Hospital of Xiamen University-ICMRS Collaborating Center for Skeletal Stem Cell, State Key Laboratory of Cellular Stress Biology, School of Medicine, Faculty of Medicine and Life Sciences, Xiamen University, Xiamen 361005, China; Xiamen Key Laboratory of Regeneration Medicine, Fujian Provincial Key Laboratory of Organ and Tissue Regeneration, Organ Transplantation Institute, School of Medicine, Xiamen University, Xiamen 361102, China; The First Affiliated Hospital of Xiamen University-ICMRS Collaborating Center for Skeletal Stem Cell, State Key Laboratory of Cellular Stress Biology, School of Medicine, Faculty of Medicine and Life Sciences, Xiamen University, Xiamen 361005, China; Xiamen Key Laboratory of Regeneration Medicine, Fujian Provincial Key Laboratory of Organ and Tissue Regeneration, Organ Transplantation Institute, School of Medicine, Xiamen University, Xiamen 361102, China; Department of Pathology and Laboratory Medicine, Weill Cornell Medical College, New York, NY 10065, USA; Research Division, Hospital for Special Surgery, New York, NY 10065, USA; The First Affiliated Hospital of Xiamen University-ICMRS Collaborating Center for Skeletal Stem Cell, State Key Laboratory of Cellular Stress Biology, School of Medicine, Faculty of Medicine and Life Sciences, Xiamen University, Xiamen 361005, China; Xiamen Key Laboratory of Regeneration Medicine, Fujian Provincial Key Laboratory of Organ and Tissue Regeneration, Organ Transplantation Institute, School of Medicine, Xiamen University, Xiamen 361102, China

**Keywords:** skeletal stem cells, bone development, endochondral ossification, intramembranous ossification, lineage tracing

## Abstract

Tissue-resident stem cells are essential for development and repair, and in the skeleton, this function is fulfilled by recently identified skeletal stem cells (SSCs). However, recent work has identified that SSCs are not monolithic, with long bones, craniofacial sites, and the spine being formed by distinct stem cells. Recent studies have utilized techniques such as fluorescence-activated cell sorting, lineage tracing, and single-cell sequencing to investigate the involvement of SSCs in bone development, homeostasis, and disease. These investigations have allowed researchers to map the lineage commitment trajectory of SSCs in different parts of the body and at different time points. Furthermore, recent studies have shed light on the characteristics of SSCs in both physiological and pathological conditions. This review focuses on discussing the spatiotemporal distribution of SSCs and enhancing our understanding of the diversity and plasticity of SSCs by summarizing recent discoveries.

## Introduction

Bone homeostasis is primarily regulated by the balance between osteoblastic bone formation and osteoclastic bone resorption (Compston et al., 2019; Méndez-Ferrer et al., 2010). While bone-resorbing osteoclasts originate from hematopoietic stem cells (HSCs) or yolk sac progenitors, bone-forming osteoblasts were traditionally viewed as derived from a group of cells termed mesenchymal stem cells (MSCs) (Bianco, 2014; Eggenhofer et al., 2014; Jacome-Galarza et al., 2019; Yahara et al., 2020). However, MSCs exhibit significant heterogeneity, being comprised of multiple cell types in unfractionated cultures (McLeod and Mauck, 2017; Tikhonova et al., 2019). Additionally, MSCs have yet to be fractionated to identify a subpopulation displaying formal stemness characteristics such as self-renewal or *in vivo* multipotency. Over the last few years, the concept of MSCs has been supplanted by better-defined cellular populations that indeed display formal stemness characteristics, frequently termed skeletal stem cells (SSCs), to distinguish these cells from prior work on MSCs (Chan et al., 2015, 2018). Furthermore, very recent work has found that SSCs are not a monolithic population, but instead are comprised of a series of related but distinct cell types that serve as stem cells at different skeletal sites or at different stages of bone development (Ambrosi et al., 2021b; Bok et al., 2023; Mizuhashi et al., 2018; Sun et al., 2023). This diversity of SSC types contributes to the distinct characteristics observed during bone development. Previously, it was known for over 300 years since the early anatomic studies of two main ways of bone formation, endochondral ossification, and intramembranous ossification (Newton et al., 2019). The process of endochondral ossification of SSCs differentiate into bone varies at different stages of bone development (Li et al., 2022; Mizuhashi et al., 2018). SSCs involved in intramembranous ossification also possess unique characteristics (Debnath et al., 2018). Recently, vertebral SSCs (vSSCs) have been identified and found to exhibit distinct characteristics compared to SSCs found in long bones and calvaria (Sun et al., 2023). This review summarizes the characteristics of SSCs in different skeletal regions and at various stages of bone development, while also exploring the overall role of SSCs in bone repair and diseases.

## Current identification and isolation methods for SSC research

While the methods to identify SSCs have a longer history in skeletal biology research, the work of Chan and colleagues to define murine and human SSCs has crystallized these methods into a key template for contemporary SSC research (Chan et al., 2015, 2018). Central to SSC research is determining the identity and differentiation trajectory of SSCs. This information is then integrated to understand the changes that occur in SSCs during bone development and with skeletal disease processes and to identify the key factors that influence the differentiation and self-renewal of these SSCs.

**Figure 1.**
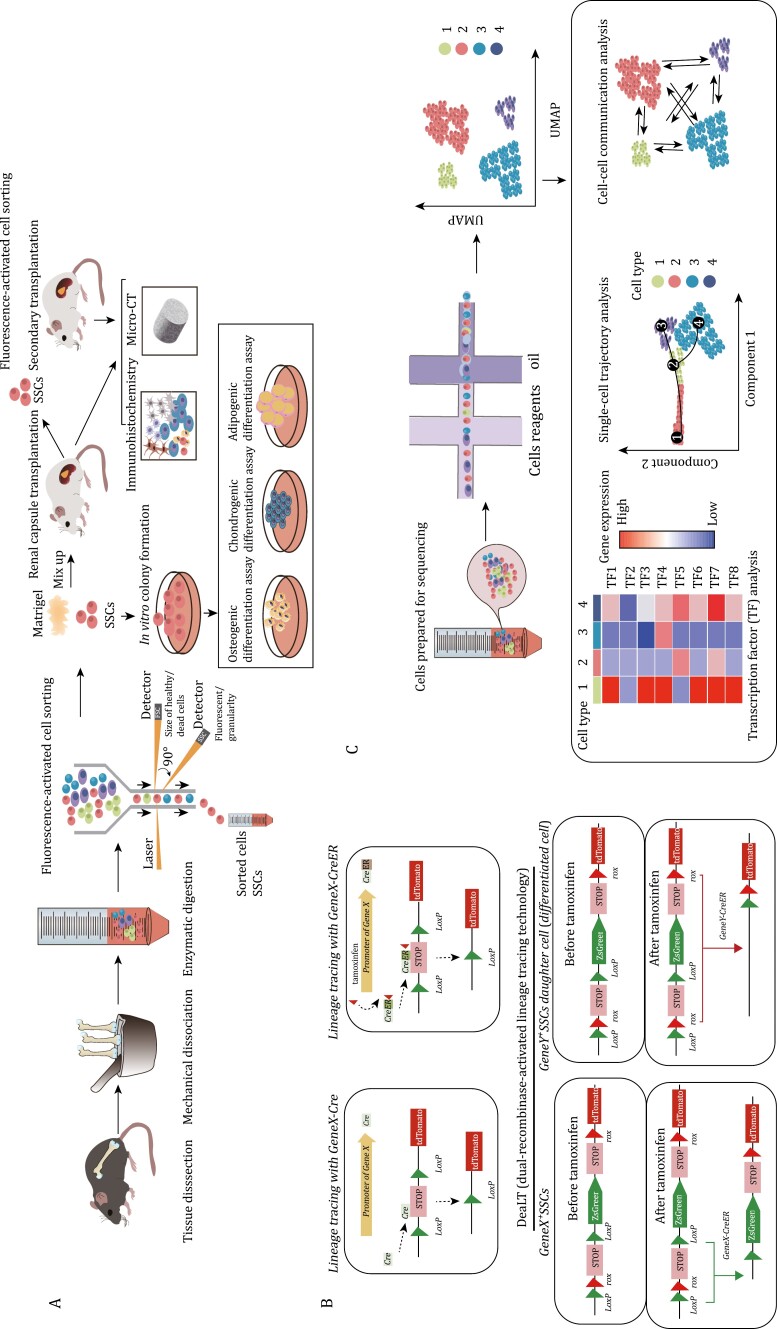
Current research methods and verification strategies on SSCs. (A) Isolation and functional assessment of SSCs: SSCs are isolated from bone tissue through mechanical grinding and enzymatic digestion, followed by sorting using FACS. The stemness and differentiation potential of SSCs are verified through *in vivo* dissimilation osteogenesis. Three-lineage differentiation experiments are conducted to confirm their differentiation ability. (B) Lineage tracing experiment: Cre recombinase is used to express the reporter gene (e.g., tdTomato). This allows permanent marking of cells expressing target gene and their progeny cells. In the CreER system, tdTomato expression is induced only when tamoxifen is administered. By giving tamoxifen at different time points, cre-expressing cells and their progeny can be permanently labeled. The DeaLT tracking system enables labeling of two different types of cells simultaneously. After tamoxifen induction, lineages can be distinguished by being labeled with different fluorescence by different genes. (C) Flow chart of single-cell sequencing and data analysis involves various analyses such as cell subtype classification, transcription factor analysis, single-cell trajectory analysis, and cell–cell communication.

The isolation of SSCs primarily relies on cell surface markers (Gulati et al., 2018). Currently, the main approach to define SSCs and the downstream cellular populations they produce is fluorescence-activated cell sorting (FACS), often used in combination with a *Cre-loxP*-driven lineage tracing strategy (Gulati et al., 2018; Kretzschmar and Watt, 2012) (Fig. 1A and Table 1). FACS combined with *in vivo* transplantation serves as the current gold standard for evaluating the differentiation potential of SSCs (Gulati et al., 2018). In this method, a small number (103–105) of cells or *ex vivo* cultured cells are obtained by FACS and mixed with a small amount of Matrigel (2–10 μL) and then transplanted into the renal subcapsular space (Gulati et al., 2018). Alternatively, similar grafts can also be implanted into intramuscular sites or the inguinal fat pad (Bok et al., 2023; Sun et al., 2023). After 2–8 weeks, ectopic bones that are often termed *in vivo* bone organoids or spicules are formed that can be analyzed by either histology or sorted by FACS (Gulati et al., 2018). The purpose of the FACS analysis is two-fold. First, it allows for the determination of which populations as defined by cell surface markers are produced by each input population. In this manner, a differentiation hierarchy can be constructed to determine which cell types each of the FACS-defined populations are able to produce, especially if the downstream non-stem populations are also transplanted to demonstrate that they produce a progressively more restricted subset of cells within the overall lineage. This analysis allows ordering of surface marker-defined cell types from those at the apex (typically the stem cell) to those at the base (typically mature populations such as osteoblasts) in the differentiation hierarchy. In addition to this analysis, these organoid assays are also used to demonstrate SSC self-renewal by demonstrating that cells with the same surface immunophenotype as the input population remain in the organoid. Ideally, this analysis is coupled with a dye dilution proliferation method, which allows the determination that the input populations persisting at the assay endpoint underwent proliferation and therefore self-renewal.

**Table 1.** Methods for identification and isolation of SSCs.

Methods	Application	Features	References
Fluorescence-activated cell sorting (FACS)	Isolation and collection of SSCs	Differentiation hierarchy can be constructed to determine which cell type is stem cell	(Gulati et al., 2018)
Methods of cultured SSCs *in vitro*	Colony-forming unit-fibroblast (CFU-F) method	Assess CFU-F activity, as a secondary method for identifying SSCs	(Bianco and Robey, 2015)
	*In vitro* tri-lineage differentiation assays	Assess the multilineage differentiation potential of SSCs	(Gulati et al., 2018)
*In vivo* transplantation	Transplanted into the renal subcapsular space, intramuscular sites or the inguinal fat pad	FACS combined with *in vivo* transplantation serves as the current gold standard for evaluating the differentiation potential of SSCs	(Bok et al., 2023; Sun et al., 2023)
Lineage tracing	*Cre-lox*P systems	Tracking the fate of SSCs; labeling multiple cell states within a lineage	(Mizuhashi et al., 2018)
	*Dre-rox* and *Flp-frt* systems		
	DeaLT systems		
Single-cell RNA sequencing (ScRNA-seq)	To study the cell heterogeneity and development process	To study cellular composition; cell heterogeneity and cell transcriptome analysis	(Debnath et al., 2018; Greenblatt et al., 2019)

Generally *in vitro* methods are best considered as secondary supporting methods in studying or defining SSC populations. SSC proliferative capacity can be assessed *in vitro* using the colony-forming unit-fibroblast (CFU-F) method (Bianco and Robey, 2015). However, CFU-F activity is widely distributed among both stem and non-stem populations and is, therefore, of questionable utility in defining SSCs outside of use as a secondary supporting methodology. Serial mesensphere formation capacity under ultra-low adherence conditions may be a more reliable correlate of stemness, but even then should only be considered a secondary supporting method (Bok et al., 2023; Chan et al., 2018; Gulati et al., 2018; Sun et al., 2023). *In vitro* tri-lineage differentiation assays assess the multilineage differentiation potential of SSCs under different culture and induction conditions, including differentiation into osteoblasts, chondrocytes, and adipocytes, however many non-stem skeletal cell types show *in vitro* tri-lineage differentiation potential (Gulati et al., 2018; Pittenger et al., 1999).

While it is crucial to assess the stemness and viability of SSCs through *in vivo* transplantation and *in vitro* cell culture, these approaches cannot replace the assessment of SSC activity in the native skeletal environment (Gulati et al., 2018). As a result, lineage tracing has become a core approach for studying SSCs, particularly in tracking the fate of these cells (Kretzschmar and Watt, 2012; Newton et al., 2019; Worthley et al., 2015). The most commonly used system for lineage tracing is *Cre-lox*P (Feil et al., 2009) (Fig. 1B). This system relies on the use of *Cre* or *CreER* recombinase. *CreER* recombinase can be induced by tamoxifen administration, which activates the expression of a specific target gene. The tamoxifen-induced *CreER* expression system allows for pulse labeling one or more lineages of cells (Mizuhashi et al., 2018). However, it should be noted that, with a few exceptions, often *CreER* expression is not restricted to a single-cell state or cell type, imposing a strong limitation in *CreER*-based approaches that necessitate the use of transplantation-based systems that are capable of resolving the functions and differentiation potential of specific cell types within a lineage. In addition to labeling multiple cell states within a lineage, many skeleton-relevant cre lines may further co-label multiple independent lineages of skeletal cells, which is a further limitation (Bok et al., 2023).

The *Dre-rox* and *Flp-frt* systems are additional methods utilized for lineage tracing in mouse models (Kretzschmar and Watt, 2012). *Dre-rox system* exhibits high recombination efficiency and does not cross-react with Cre recombinase. These two systems can be combined to achieve dual genetic manipulation (He et al., 2018). Lineage tracing often involves the use of fluorescence reporter mice. The Rosa26 site is currently one of the most commonly employed sites for site-directed integration of fluorescent reporters (Soriano, 1999). By crossbreeding Rosa26 site-integrated fluorescence reporter mice with different Cre recombinase-expressing strains, researchers can conduct tissue-specific cell lineage tracing (Feil et al., 2009; McLellan et al., 2017). Many well-known SSC markers are not fully specific to only a particular SSC type and are often co-expressed by multiple lineages of cells. This poses a challenge in tracking the fate and origin of SSCs. To address this problem, DeaLT (dual-recombinase-activated lineage tracing technology) incorporates the *Dre-rox* recombination system into the traditional *Cre-loxP* recombination system and can be a effective solution (He et al., 2018). By utilizing one recombination system (*Dre-rox*) to control another system (*Cre-loxP*), the *Dre-rox*-mediated recombination reaction removes the *loxP* site in cells that may cause ectopic expression of *Cre* (He et al., 2018). This effectively prevents ectopic recombination of *Cre-loxP* and enhances the accuracy of *Cre-loxP*-mediated lineage tracing results (He et al., 2018). DeaLT also offers a method to track two different cell populations simultaneously (He et al., 2018).

Single-cell RNA sequencing (ScRNA-seq) can be used to study cellular composition, though the sparse transcriptional sampling inherent in the technique, often termed “dropout,” is likely to lead to an inability to resolve the full set of biologically distinct cell states present unless specialized techniques are utilized (Greenblatt et al., 2019; Jin et al., 2023). ScRNA-seq has also contributed to the understanding of SSCs, such as sorting SSCs and other skeletal lineage cells, and analyzing the signaling pathways that may regulate the activity of SSC and its progeny (Chan et al., 2015). Lineage tracing technology, when combined with single-cell sequencing, can be utilized to analyze the heterogeneity of cells derived from target gene-marked lineages (Debnath et al., 2018; Kretzschmar and Watt, 2012). For instance, CTSK-lineage cells were sorted for single-cell sequencing, revealing the presence of four subpopulations, including PSCs (Debnath et al., 2018). Additionally, single-cell trajectory analysis can provide evidence for the differentiation trajectory of SSCs, though it is considered essential that any computationally inferred differentiation trajectories be supported by direct experimental studies. By enabling the discovery of key regulatory factors and cellular interactions, ScRNA-seq technology has greatly advanced SSC research (Chan et al., 2015; Debnath et al., 2018) (Fig. 1C).

## SSCs in long bone at different bone development stages

### Emergence of embryonic skeletal progenitors and formation of primary ossification center

**Figure 2.**
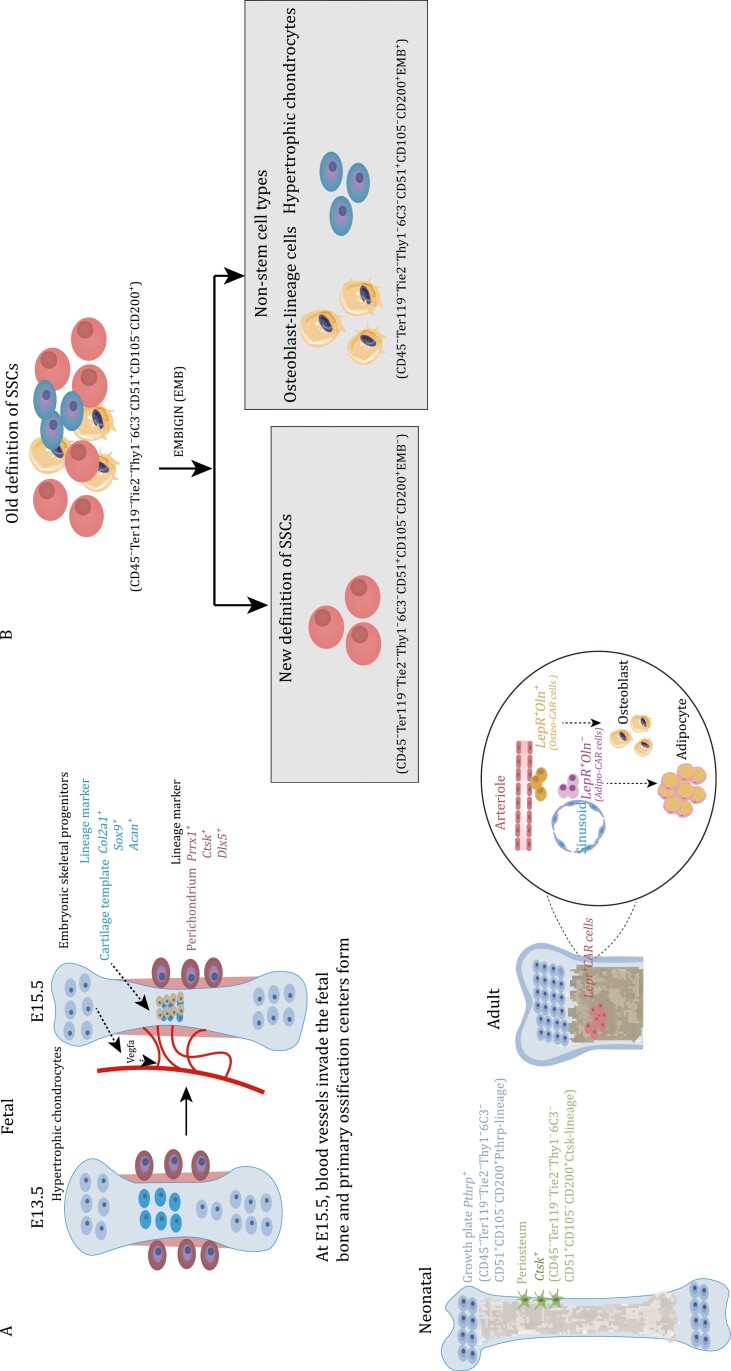
Marker genes of embryonic skeletal progenitors and SSCs at different time points in long bones and the current definition of SSCs immunophenotype. (A) At E15.5, hypertrophic chondrocytes secrete Vegfa to induce blood vessel invasion and form the primary ossification center. Embryonic cartilage templates are labeled by *Col2a1*, *Sox9*, and *Acan*. Perichondrium is labeled by *Prrx1*, *Hox11*, *Ctsk*, and *Dlx5*. Postnatal SSCs in the growth plate are labeled by *Pthrp.* Periosteal SSCs are labeled by *Ctsk*. *Lepr*^*+*^ CAR cells can be distinguished into osteogenic *Oln*^*+*^ cells next to the arteriole and adipogenic Oln^−^ cells next to the sinusoid. (B) The previous immunophenotype of SSCs was defined as Lin^−^CD200^+^CD105^−^THY1^−^6C3^−^, which included osteoblasts and hypertrophic chondrocytes. The EMB marker can distinguish the osteoblasts and hypertrophic chondrocytes mixed into SSCs, and the updated immunophenotype of SSCs is now defined as Lin^−^CD200^+^CD105^−^THY1^−^6C3^−^EMB^−^.

The limb bud starts to develop around embryonic day (E) 9.5 (forelimb) or E10.5 (hindlimb). By E15.5, hypertrophic chondrocytes release vascular endothelial growth factor A (VEGFA) to facilitate vascular invasion, leading to the formation of the primary ossification center (Gerber et al., 1999) (Fig. 2A and Table 2). As fetal development progresses, the cartilage template is gradually replaced by bone (Zeller et al., 2009). It is believed that the early osteoblasts originate from fetal cartilage and fetal perichondrium (Arostegui et al., 2022). Recent studies have revealed that chondrocytes do not uniformly undergo programmed cell death, but instead, a subset may transform into osteoblast lineage cells and contribute to the formation of the primary ossification center (Yang et al., 2014; Zhou et al., 2014b). Fetal cartilage expresses chondrocyte-specific marker genes, such as *Type II collagen alpha 1 chain* (*Col2a1*), collagen, type XI, alpha 2 (*Col11a2)*, and *Aggrecan* (*Acan*), as well as *SRY-box transcription factor 9* (*Sox9*) (Ng et al., 1997; Zhao et al., 1997). *Sox9* is expressed in chondrocytes, except for hypertrophic chondrocytes (Zhao et al., 1997). Cell fate mapping of *Sox9*^*+*^ cells in early limb development from E 8.0 to E17.0 suggests that all osteo-chondroprogenitor cells are derived from *Sox9*-expressing precursors (Akiyama et al., 2005). *In vivo* lineage tracing studies using *Acan-creER* and *Col2a1-creER* mice similarly indicate that long-lived, likely multipotent, progenitor populations are marked by these traditionally chondrocyte-associated genes (Ono et al., 2014; Zhou et al., 2014b).

**Table 2.** SSCs and skeletal progenitors labeled by lineages.

Gene	Transgenes	Location	Features	References
*Col2a1*	*Col2a1-Cre*	Fetal cartilage, long bone	Multipotent, progenitor populations	(Zhao et al., 1997)
*Acan*	*Acan -Cre*	Fetal cartilage; long bone growth plate	Primary source of osteoblasts during adolescence	(Shu et al., 2021)
*Sox9*	*Sox9-Cre* *Sox9-CreERT*	Fetal cartilage	All osteo-chondroprogenitor cells	(Shu et al., 2021)
*Dlx5*	*Dlx5-CreER*	Perichondrium	Early marker for the perichondrium	(Matsushita et al., 2022)
*Osx*	*Osx-Cre* *Osx-CreER*	Fetal perichondrium, growth cartilage	Osx labeled cells are transient	(Mizoguchi et al., 2014)
*Ctsk*	*CTSK-mGFP* *Ctsk-Cre*	Long bone and calvarial Periosteum	Label pscs	(Bok et al., 2023; Debnath et al., 2018)
*PTHrP*	*PTHrP-mCherry* *PTHrP-CreER*	Long bone growth plate	Located in the resting zone of the postnatal growth plate	(Mizuhashi et al., 2018)
*LepR*	*LepR-Cre* *LepR-CreER*	Bone marrow	Primary source of cxcl12	(Zhou et al. 2014a)
*Zic1*	*Zic1- Cre*	Vertebra	Label vsscs	(Sun et al., 2023)
*Pax1*	*Pax1-creER* ^*T2*^	Vertebra	Label vsscs	(Sun et al., 2023)
*Gli1*	*Gli1-CreERT*	Growth plate; craniofacial bones	Widespread and encompass numerous non-ssc populations	(Shi et al., 2017)
*Prrx1*	*Prx1-Cre*	Long bones	Labeling almost all skeletal lineage cells in long bones	(Liu et al., 2022)

The fetal perichondrium surrounds fetal cartilage templates and consists of several layers of elongated fibroblasts (Matsushita et al., 2022). *Osterix* (*Osx*)-expressing cells are found in the innermost layer of the perichondrium and were labeled in the perichondrium before the formation of the medullary cavity at E13.5 (Maes et al., 2010; Ono et al., 2014). Upon invasion of blood vessels, the Osx-expressing cells rapidly expand and enter the developing marrow space (Maes et al., 2010; Mizoguchi et al., 2014; Ono et al., 2014). Over time, these cell groups and their descendants gradually differentiate into osteoblasts, leading to the formation of the primary ossification center (Mizoguchi et al., 2014). Ono et al. (2014) conducted a study in which they mapped the fates of *Osx*^+^ cell and *Col2*^+^ cells, finding that that *Osx*^+^ cells in the perichondrium and the marrow space originate from *Col2*^*+*^ cells. This *Col2a1*, *Acan*, *Sox9*, and other chondrocyte markers are likely to broadly label SSCs in addition to labeling chondrocytes, though at least certain SSC populations, such as growth plate resident *PTHrP*^*+*^ cells may have a chondrocytes identity themselves (Mizuhashi et al., 2018). Previous reports have shown that *Prrx1-Cre* marks all mesenchymal cells at E9.5, and these *Prrx1*^*+*^ cells possess the potential to differentiate into chondrocytes, perichondrial cells, periosteal cells, osteoblasts, and stromal cells, excluding muscle cells (Logan et al., 2002). *Hox11* is tightly expressed in the zeugopod perichondrium in *Sox9-*positive chondrocytes, as well as in *Osx-*expressing cells (Pineault et al., 2019). Additionally, *Cathepsin K* (*Ctsk*), previously known as a mature osteoclast marker, has been found to label a periosteal SSC lineage (PSCs). *Ctsk*^*+*^ periosteal cells were first observed on the perichondrium at E14.5 (Debnath et al., 2018). Although Prrx1-creER, Hoxa11-creER, Sox9-creER, and Axin2-creER have been used to track cell populations on cartilage templates or perichondrium during embryonic stages, it remains uncertain whether these cell populations overlap SSCs (Xie et al., 2022). Matsushita et al. (2022) utilized FACS to sort *Col2a1*^*+*^ cells for scRNA-seq analysis and discovered that *Dlx5* can serve as an early marker for the perichondrium. Dlx5-lineage cells primarily contribute to the cortical bone and marrow stromal compartments instead of generating cartilage (Matsushita et al., 2022).

The transcriptional landscape of human limb bud and embryonic long bone was mapped using single-cell sequencing at 5 weeks and 8 weeks post conception (He et al., 2021). The study reveals a specific group of perichondrial progenitors which exhibit strong expression of the adhesion molecule CADM1 and are enriched for the FOXP1/2 transcriptional network (He et al., 2021). Notably, FOXP1/2+ perichondrial progenitors possess the ability to coordinate both endochondral and intramembranous ossification processes, making them pivotal in embryonic development (He et al., 2021). Similar to the fetal cartilage template, the early perichondrial cells have been shown to contribute to the generation of osteoblasts. Although current evidence suggests that fetal cartilage template and perichondrial cells are important sources of osteoblasts, it is still unclear whether there are unique cell populations of embryonic SSCs. Some genes, such as Col2a1, have been confirmed to broadly label SSCs. Additionally, the cartilage template is gradually replaced, but the articular cartilage is not. This raises the question of whether fetal chondrocytes located at a specific location have different SSCs. However, further confirmation regarding this question is still required. Further confirmation is necessary to determine the exact source of the embryonic skeletal progenitors’ group that contributes to early bone formation.

### Neonatal SSCs during long bone growth

Following birth, bone undergo a phase of rapid growth, coinciding with the activation of SSCs. Fate-tracing experiments have demonstrated that certain cell types primarily contribute to the process of osteogenesis. Tracing the fate of skeletal progenitor cells using *Col2-CreER*; *R26R-tdTomato* mice, who received tamoxifen injection at P3, reveals that Col2-lineage cells harbor the ability to generate metaphyseal and epiphyseal osteoblasts and stromal cells (Ono et al., 2014). Similarly, populations of osteoblasts and stromal cells are also derived from *Sox9-CreER* and *Acan-CreER* (Shu et al., 2021). *Acan*^*+*^ cells in the growth plate serve as the primary source of osteoblasts during adolescence, and some of these cells also transit into Leptin receptor (*Lepr*)^+^ cells, contributing to the pool of osteoblasts (Shu et al., 2021). *Osx*^*+*^ cells are capable of labeling some skeletal progenitor cells after birth, but Osx labeled cells are transient (Mizoguchi et al., 2014). In *Osx-CreERT2*; *R26R-tdTomato* mice, Tomato^+^ cells labeled at P5 can continue to contribute to marrow stromal cells until 32 weeks (Mizoguchi et al., 2014). Lineage tracing experiments on postnatal skeletal progenitor cells have confirmed their contribution to the pool of osteoblasts. However, it is important to note that these cells not only contain SSCs but also non-stem populations. Therefore, defining the immunotype of SSCs using cell surface markers can help reduce this heterogeneity. Chan et al. conducted a systematic study of postnatal SSCs. They defined SSCs based on surface markers (CD45^−^Ter119^−^Tie2^−^Thy1^−^6C3^−^CD51^+^CD105^−^CD200^+^) through FACS and renal capsule transplantation (Chan et al., 2015). The research conducted by Chan and colleagues offers valuable insights into the field of SSC research. Their work systematically explores the immune phenotype of SSCs and effectively illustrates the characteristics and differentiation trajectories of cell populations at different stages, including SSCs and their progeny (Chan et al., 2015). However, the specific anatomical location of this group of FACS-purified SSCs is currently unclear. Ono et al. conducted a study using the knock-in *Pthrp-mCherry* reporter mice to demonstrate the formation of *PTHrP*^*+*^ chondrocytes (Mizuhashi et al., 2018). *PTHrP*^*+*^ chondrocytes were mainly located in the resting zone of the postnatal growth plate, with a significant increase in their numbers at postnatal time points P6 and P9 (Mizuhashi et al., 2018). These PTHrP-lineage chondrocytes also expressed specific markers for SSCs (Mizuhashi et al., 2018). One notable feature of long bone development after birth is the formation of a secondary ossification center (SOC) (Newton et al., 2019). SSCs also contribute to the formation of a stem cell niche after the SOC is established, allowing for self-renewal of SSCs without depletion (Newton et al., 2019). A specific group of SSCs marked by Ctsk, also characterized by CD45^−^Ter119^−^Tie2^−^Thy1^−^6C3^−^CD51^+^CD105^−^CD200^+^ and found in the periosteum, has been confirmed as periosteal stem cells (PSCs) (Debnath et al., 2018). Immunofluorescence imaging has shown that *Ctsk*^*+*^ cells, which include mature osteoclasts, also encompass SSCs, pre-bone, cartilage, and stromal progenitor cells (pre-BCSPs) and bone, cartilage, and stromal progenitor cells (BCSPs) (Debnath et al., 2018). Furthermore, it has been observed that PSCs are highly concentrated in chimeric areas within the neonatal mouse skull (Debnath et al., 2018). This indicates that their primary role is intramembranous ossification rather than endochondral ossification. Additionally, the growth plates and periosteum seem to possess a common set of surface markers of SSCs (Lin^−^THY1^−^6C3^−^CD51^+^CD200^+^CD105^−^) (Mizuhashi et al., 2018). Recent research has also provided evidence of the existence of CD200^+^CD105^−^SSCs in vertebral tissues (Sun et al., 2023). Although CD200^+^CD105^−^ SSCs have been confirmed to be at the top of differentiation in serial transplantation experiments, this population has been shown to express osteocalcin (Ocn), which is a signature marker of the osteoblastic lineage (Matthews et al., 2021). Another study has also confirmed that osteoblast lineage cells express CD200 (Takahashi et al., 2019). In order to update the current SSC definitions, a research for surface markers has confirmed that *embigin (EMB)* can be used as a marker to distinguish osteoblasts from CD200^+^CD105^−^SSCs (Sun et al., 2023) (Fig. 2B). *EMB* effectively distinguishes SSCs into two groups of cells: EMB^−^ cells and EMB^+^ cells (Sun et al., 2023). EMB^+^ cells strongly express osteoblast marker genes, including *Alpl*, *Spp1*, and *Sp7* (Sun et al., 2023). *In vitro* mammary fat pad transplantation experiments have confirmed that EMB^−^ cells can generate EMB^+^ cells, but EMB^+^ cells cannot generate EMB^−^ cells (Sun et al., 2023). Thus, EMB identifies mature cell types, such as osteoblasts and hypertrophic chondrocytes, that help refine the current stem cell immunophenotypic definitions. Therefore, the immunotype of SSCs has also been updated to Lin^−^THY1^−^6C3^−^CD51^+^CD200^+^CD105^−^EMB^−^ (Sun et al., 2023) (Fig. 2B).

### Adult SSCs in long bone maintenance and bone marrow niche

The transition of long bones from birth to adulthood primarily involves a shift from rapid bone growth to the maintenance of bone homeostasis (Clarke, 2008). LepR^+^ cells, which constitute approximately 0.3% of bone marrow cells, are responsible for the formation of almost all CFU-Fs in the bone marrow (Zhou et al., 2014a). LepR^+^ cells are the primary source of Cxcl12 (CAR cells), and these stromal cells have also been validated to originate from SSCs in an organoid assay (Bok et al., 2023; Kara et al., 2023). After adolescence, *Lepr*^*+*^ cells become the predominant source of osteoblasts (Shu et al., 2021). Adult long bones mainly consist of *Prrx1*^*+*^ cells, which encompass nearly all skeletal lineages (Yue et al., 2016). When comparing Prrx1-Cre and Lepr-Cre-traced cells in adult long bones through single-cell sequencing, the cells derived from Lepr or Prrx1 can be categorized into nine subsets, which include bone marrow stromal cells (BMSCs), pre-osteoblasts (pre-OBs), osteoblasts (OBs), chondrocytes, αSMA^+^ cells, and periosteal cells. Both traced cells marked similar numbers of BMSCs, while Prrx1-Cre primarily marks pre-OBs and chondrocytes (Mo et al., 2022; Yue et al., 2016). Additionally, apart from CAR cells found in the bone marrow cavity, there are a subpopulation of CAR cells located in the periosteal membrane that exhibit robust clonogenic and adipogenic differentiation capabilities (Jeffery et al., 2022; Mo et al., 2022). Subpopulations of CAR cells, namely Adipo-CAR cells and Osteo-CAR cells, exhibit distinct localization on the surface of sinusoids or arterioles. These cells function as specialized cytokine-secreting cells within their respective perivascular niches (Baccin et al., 2020). CAR cells in the bone marrow can be categorized into two groups based on Osteolectin (Oln) expression (Shen et al., 2021). The first group consists of periarteriolar *Oln*^*+*^ cells, which differentiate into osteoblasts (Shen et al., 2021). The other group comprises perisinusoidal *Oln*^−^ cells, which differentiate into adipocytes in homeostasis (Shen et al., 2021) (Fig. 2A). However, it is important to note that this second group of cells also plays a significant role in bone injuries and can later undergo osteogenic transition (Shen et al., 2021). In addition to their role in osteogenesis and adipogenesis, CAR cells in the bone marrow are responsible for maintaining the homeostasis of the bone marrow niche. Deletion of the periarteriolar *Oln*^*+*^ cells leads to a decrease in common lymphoid progenitors and subsequently impairs the immune response in mice (Shen et al., 2021).

## Confirmation of vSSCs and induction of spinal metastases

**Figure 3.**
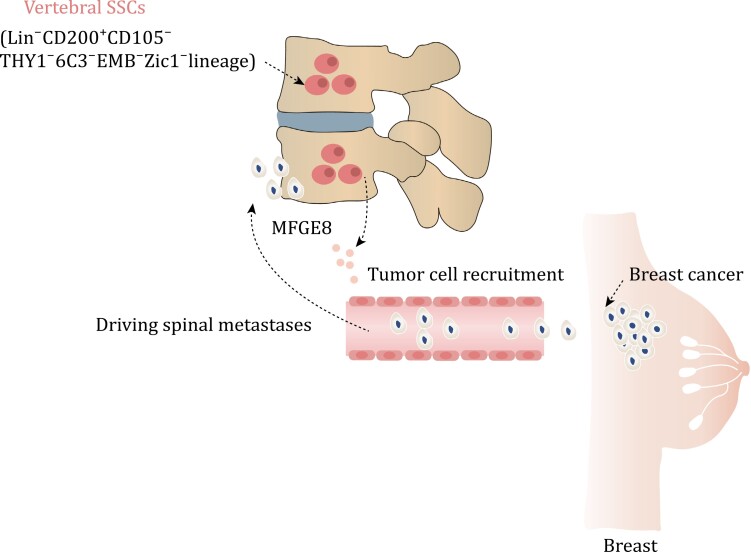
vSSCs driving spinal metastases in breast cancer. The vSSCs, which are characterized by the presence of *Zic1*, exhibit an immunophenotype of Lin^−^CD200^+^CD105^−^THY1^−^6C3^−^EMB^−^. These vSSCs secrete MFDE8, a molecule that plays a crucial role in attracting breast cancer cells and facilitating their metastasis to the spine.

*Prrx1-cre* is capable of labeling almost all skeletal lineage cells in long bones, but it does not label any skeletal lineage cells in the spine (Liu et al., 2022; Sun et al., 2023). This phenomenon suggests the possible presence of a unique SSCs in the vertebral region. In a recent study, Sun et al. utilized an immunophenotype (Lin^−^CD200^+^CD105^−^THY1^−^6C3^−^EMB^−^ cells) to analyze SSCs in the vertebra (vSSCs) (Sun et al., 2023). They found that *Zic1* and *Pax1* are highly expressed in these vSSCs (Sun et al., 2023) (Fig. 3 and Table 2). *Zic1* and *Pax1* cells can specifically label vSSCs, but not SSCs in long bones. Importantly, this group of vSSCs demonstrates the ability for *in vivo* self-renewal and the potential to differentiate into multiple cell lineages (Sun et al., 2023). Knockout of *Osx* in *Zic1*^*+*^ cells lead to a severe spinal instability, while long bone phenotype remains normal (Sun et al., 2023). Knocking out the osteogenic positive regulatory signal transducer and activator of transcription (STAT3) in *Zic1*^*+*^ cells resulted in bone mass loss, whereas knocking out the negative regulatory factor *Schnurri-3* in *Zic1*^*+*^ cells leads to increase of bone mass (Jones et al., 2007; Sun et al., 2023; Zhou et al., 2021). It is worth mentioning that the observed mouse bone phenotypes caused by both knockout experiments were specifically observed in the vertebral body (Jones et al., 2007; Sun et al., 2023; Zhou et al., 2021). It is important to note that vSSCs not only contribute to bone formation but also play a role in tumor metastasis to the spine. Upon injection of breast cancer cells into the circulation, it was observed that more tumor cells metastasized and seeded into the spine compared to the long bones (Sun et al., 2023). This difference cannot be attributed to specific features of blood vessels or the rate of blood flow but is caused by differences between SSCs in long bones and the spine (Sun et al., 2023). The ability of vSSCs to drive tumor cell metastasis has also been demonstrated using bone organoids—3D structures grown *in vitro*. This difference is also attributed to the fact that vSSCs produce high levels of the protein milk fat globule EGF (Mfge8), which effectively stimulates the migration ability of tumor cells (Sun et al., 2023). Deletion of Mfge8 can significantly reduce the tendency of tumor cells to metastasize to the spine (Sun et al., 2023). The confirmation of vSSCs may not only explain the ease of tumor metastasis, but it may also provide insight into the potential clinical correlation between other spine-related diseases and vSSCs in the future. Furthermore, future research could focus on reducing the risk of spinal cancer metastasis by blocking Mfge8.

## SSCs in calvarial mineralization and craniosynostosis

The bone formation mode of the calvaria differs significantly from long bones (Maruyama, 2019; Maruyama et al., 2021). The skull primarily relies on intramembranous ossification (He et al., 2021; Wilk et al., 2017). Additionally, the calvaria has a distinct population of SSCs different from long bones and spine (Maruyama, 2019). In long bones, *Gli1*^*+*^ cells mainly label growth plate SSCs after birth and PSCs in adulthood (Shi et al., 2017). However, *Gli1*^*+*^ cells are also expressed in the suture mesenchyme of craniofacial bones postnatally, and they contribute to craniofacial bone turnover in adulthood (Jing et al., 2022; Men et al., 2020; Zhao et al., 2015). The absence of *Gli1*^*+*^ cells results in craniosynostosis and stunted skull growth, highlighting their importance in skull development (Zhao et al., 2015). Combining *Gli1*^*+*^ cells with biodegradable materials can regenerate functional skull sutures and correct deformities (Yu et al., 2021). *Gli1*^*+*^ cells secrete vascular endothelial growth factor-C, which promotes lymphatic endothelial cell proliferation and migration (Ma et al., 2023).

**Figure 4.**
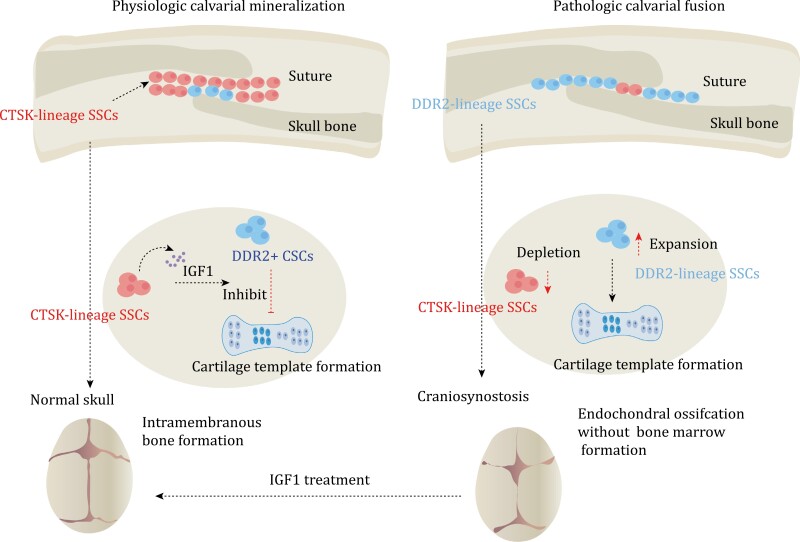
SSCs in calvarial mineralization and craniosynostosis. Calvaria SSCs are labeled by *Ctsk.* Under normal conditions, *Ctsk^+^* SSCs secrete IGF1 to inhibit endochondral ossification process of *DDR2^+^* SSCs. The process mainly relies on intramembranous ossification, which is dominated by *Ctsk^+^* SSCs. However, in pathologic calvarial fusion, *Ctsk^+^* SSCs become depleted. This depletion leads to the activation of the endochondral ossification process of *DDR2^+^* SSCs, resulting in the occurrence of craniosynostosis. In mice, the administration of IGF1 has been shown to rescue the occurrence of craniosynostosis.

However, *Gli1*^+^ cells are widespread and encompass numerous non-SSC populations (Shi et al., 2017). On the other hand, *Prrx1* is present in almost all skeletal lineage cells in the long bone marrow, but it is limited to the calvarial suture niche in the skull and decreases with age (Wilk et al., 2017). However, the loss of this population of *Prrx1*^*+*^ cells does not impact skull development. Therefore, unlike long bones, the population of *Prrx1*-labeled cells in the skull is restricted (Wilk et al., 2017). In long bones, *Ctsk*^*+*^ cells are primarily located in the periosteum and play a crucial role in driving intramembranous ossification (Debnath et al., 2018). These cells are also found enriched in the sagittal suture and skull periosteum (Bok et al., 2023; Debnath et al., 2018). When the gene *Twist1* that is mutated in Saethre-Chotzen syndrome is knocked out in *Ctsk*-lineage cells, it recapitulates the signature craniosynostoiss phenotype associated with *Twist1* loss (Bok et al., 2023; Maruyama, 2019; Yu et al., 2021). Interestingly, contrary to expectations, the premature fusion of the skull did not lead to an expansion of *Ctsk*-lineage cells. Instead, the Ctsk-lineage cells were depleted and replaced by *DDR2* (*discoidin domain-containing receptor 2*) lineage cells (Bok et al., 2023) (Fig. 4 and Table 2). *Twist1*^fl/fl^ Ctsk-cre mice demonstrate ectopic endochondral ossification in the cranial sutures and the ectopic bone is enriched in *DDR2*^*+*^ cells (Bok et al., 2023). Renal capsule transplantation showed that this group of *DDR2*^*+*^ cells also displayed unique osteogenesis characteristics, with no formation of a bone marrow cavity (Bok et al., 2023). Therefore, under physiological conditions, *Ctsk*^*+*^ cells are responsible for the mineralization and fusion of the skull. In the absence of *Ctsk*^*+*^ SSCs, *DDR2*^*+*^ SSCs differentiate into chondrocytes, contributing to the observed suture fusion (Bok et al., 2023). Both *Ctsk*^*+*^ cells and *DDR2*^*+*^ cells express *Gli1*, *Axin2*, and *Prrx1*, suggesting a partial overlap between *Gli1*, *Axin2*, and *Prrx1*-positive SSCs and *Ctsk*^*+*^ cells and DDR2^+^ cells (Bok et al., 2023; Maruyama, 2019; Wang et al., 2022; Wilk et al., 2017; Yu et al., 2021). *Ctsk*^+^ SSCs can produce insulin-like growth factor-1 (IGF1) to regulate *DDR2*^*+*^ SSCs (Bok et al., 2023). Injection of recombinant IGF1 into *TWIST1* knockout mice effectively prevents premature skull fusion (Bok et al., 2023). Therefore, SSCs are more complex than previously thought, and skull SSCs exhibit significant differences under physiological and pathological conditions. This suggests that SSCs in other bones may also exhibit substantial differences under different conditions.

## Features of aging SSCs

**Figure 5.**
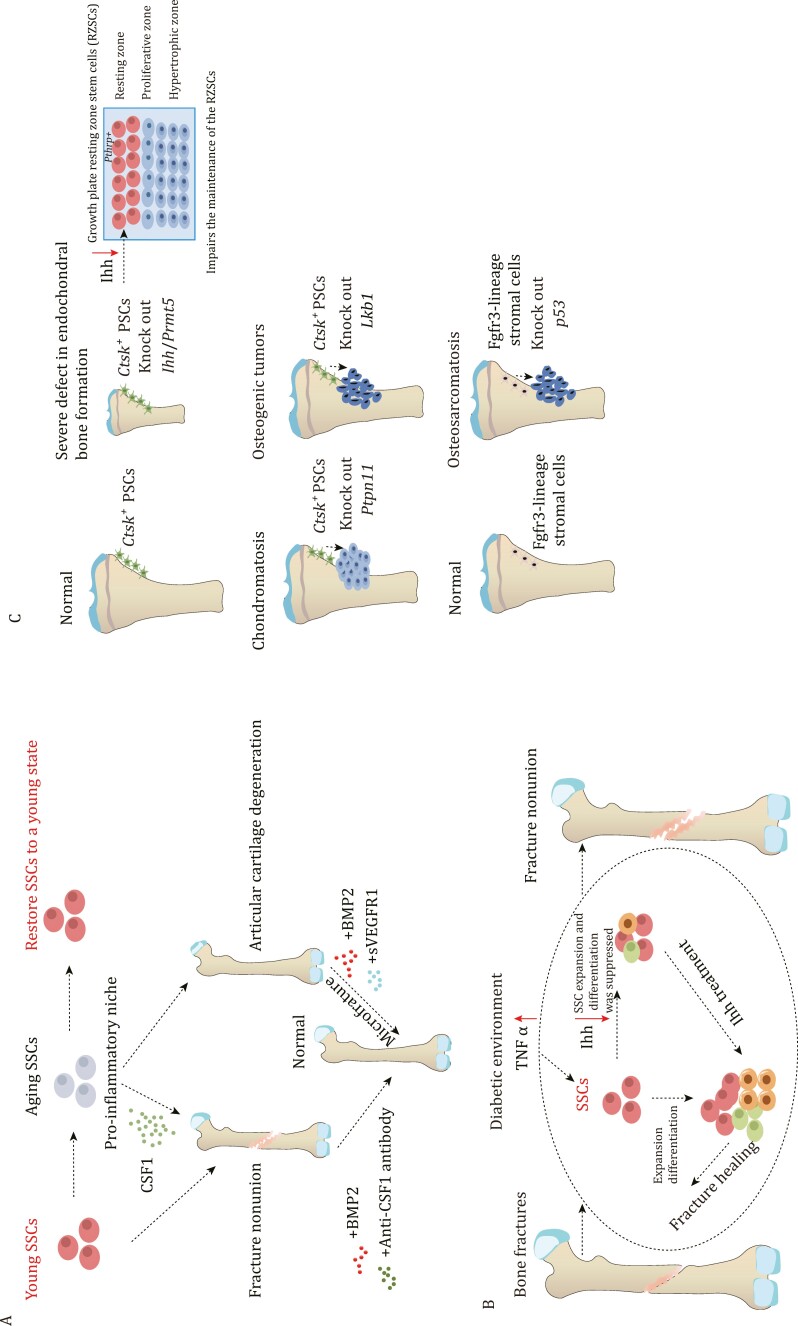
The role of SSCs in bone fracture and bone disease. (A) Aging SSCs can cause a pro-inflammatory niche through secreting CSF1, which leads to poor fracture healing. However, administration of BMP2 and anti-CSF1 antibody improves the healing process. Aging SSCs also lead to articular cartilage degeneration, but administration of BMP2 and sVEGFR1 can help improve this condition. (B) during bone fracture healing, SSCs undergo expansion and enhance their ability to differentiate in order to facilitate the repair process. However, in a diabetic environment, inflammatory signaling pathways are activated, leading to increased secretion of TNFα. This excessive TNFα inhibits Ihh, which, in turn, affects the expansion and differentiation capability of SSCs. Ihh treatment is administered to enhance the vitality of SSCs and improve their ability to repair fractures in the diabetic environment. (C) Mice with Prmt5 knockout in *Ctsk*^*+*^ PSCs display gradual impairments in both intramembranous and endochondral bone formation. The deficiency of Ihh derived from PSCs is responsible for the latter. The deletion of *Ctsk*^*+*^ PSCs-specific Ihh hampers the maintenance of resting zone stem cells, leading to significant abnormalities in postnatal endochondral bone formation. Knocking out *Ptpn11* in *Ctsk*^*+*^ PSCs leads to chondromatosis, while lack of *Lkb1* leads to osteosarcoma. Additionally, loss *p53* in endosteal Fgfr3-lineage stromal cells leads to osteosarcoma.

The occurrence of bone diseases is closely related to aging, but the aging mechanism of SSCs is still unclear. Compared to young SSCs, aging SSCs display reduced activity and loss of SSC diversity (Ambrosi et al., 2021a; Josephson et al., 2019; Murphy et al., 2020). Exposure of aged mice to young circulation through heterochronic parabiosis or systemic reconstitution with young HSCs does not reverse the reduction of osteochondrogenic activity of aged SSCs or improve bone mass and bone healing parameters (Ambrosi et al., 2021a). This suggests that the intrinsic senescence of SSCs is the underlying cause of their reduced activity (Ambrosi et al., 2021a). Furthermore, aging SSCs contribute to creating a pro-inflammatory niche in the bone marrow cavity, leading to aging-related myeloid deviation in HSCs, and promoting osteoclast activation (Ambrosi et al., 2021a). The aging of SSCs leads to a shift in lineage differentiation, which further skews the HSC and results in an increased number of myeloid cell lineages (Ambrosi et al., 2021a). There is also evidence that this aging associated SSC dysfunction may be reversible, as treatment with Bone Morphogenetic Protein-2 (BMP2) and a low-dose CSF1 antagonist restores the fracture healing capacity and mechanical strength of 24-month-old mice, and significantly enhances the osteogenic and clone-forming abilities of SSCs (Ambrosi et al., 2021a) (Fig. 5A). Changes in the bone marrow niche due to aging result in the loss of HSCs and SSCs and the alteration of the differentiation fate of stem cells (Ambrosi et al., 2021a; Mitchell et al., 2023). The aging of bone marrow is a complex process that involves the interaction between aging SSCs and HSCs (Mitchell et al., 2023). The bone marrow niche, consisting of immune cells and endothelial cells, plays a crucial role in maintaining stem cells in inflammatory degenerative environment (Ambrosi and Chan, 2023; Mitchell et al., 2023). However, current research on the niche of SSCs is not fully understood. Therefore, regulation of the SSCs niche and restoring the optimal state of SSCs is a significant area for future research.

## Role of SSCs in skeletal repair and disease processes

Similar to the bone development process, the fracture repair process also involves both endochondral and intramembranous ossification (Allen et al., 2004; He et al., 2017). The number of SSCs significantly increases after bone fracture, and SSCs that are isolated from fracture callus also show an enhanced capacity for bone formation (Chan et al., 2015) (Fig. 5B). The periosteum plays a crucial role in fracture healing, for instance, osteoblasts derived from PSCs expand in response to the injury and are essential for the healing process. This was supported by the findings from *Osx*^*fl/fl*^; *Ctsk-cre* mice, which showed significantly impaired fracture healing, indicating the indispensable role of PSC-derived osteoblasts in the healing process (Debnath et al., 2018). Furthermore, it has been observed that obesity and type 2 diabetes significantly hinder the fracture healing ability (Khosla et al., 2020; Tevlin et al., 2017). It was found that compared to non-diabetes mellitus mice, the ability of fracture healing was reduced in the Diabetes mellitus mice. The impaired fracture healing ability is due to a significant reduction in the expansion of SSCs and BCSPs at the fracture sites (Tevlin et al., 2017). Additionally, the elevated level of TNFα caused by Diabetes mellitus systemically inhibits the expression of Indian hedgehog (Ihh) signaling, which suppresses the injury-induced expansion of mSSCs during fracture repair, leading to impaired healing (Tevlin et al., 2017). In contrast to the strong regenerative ability of bone, articular cartilage damage is often considered irreversible (Armiento et al., 2019). Nevertheless, in the microfracture model, SSCs have demonstrated the potential to regenerate articular cartilage with BMP2 application, replacing it with functional cartilage rather than fibrocartilage (Murphy et al., 2020). This restoration of cartilage has the potential to improve the mobility of osteoarthritis mice (Murphy et al., 2020). PSCs play a crucial role not only in intramembranous osteogenesis but also in endochondral ossification (Tsukasaki et al., 2022). *Prmt5*^fl/Δ^; Ctsk-Cre mice show bone growth defects, and the absence of PSCs significantly impacts endochondral bone formation (Tsukasaki et al., 2022). This abnormal bone growth is primarily attributed to the deficient secretion of Ihh by PSCs (Tsukasaki et al., 2022). Ihh is essential for bone growth, as evident from the noticeable bone growth impairment observed in *Ihh*^fl/fl^; Ctsk-Cre mice (Tsukasaki et al., 2022). These findings underscore the importance of *Ctsk*^*+*^ PSCs in both intramembranous ossification and endochondral ossification (Fig. 5C). SSCs also play a significant role in bone tumorigenesis. Knockout of *Ptpn11* in Ctsk-lineage cells leads to chondromatosis (Yang et al., 2013). Moreover, Ctsk-lineage cells with *liver kinase b1*(*LKB1*) deletion exhibit a substantial increase in cell numbers and an enhanced ability to differentiate into osteoblasts (Han et al., 2019). Knockout mice of *LKB1* in Ctsk-lineage cells result in tumor formation in the periosteum, primarily due to the activation of the mTORC1 pathway (Han et al., 2019). Similarly, the knockout of *p53* in endosteal Fgfr3-lineage stromal cells also leads to osteosarcoma with transcriptional features similar to that of human chondroosteosarcoma (Matsushita et al., 2023). A study aims to analyze osteosarcomas using immunohistochemistry to identify a specific subset of osteosarcoma expressing Lepr, indicating that CAR cells are the origin for this particular subset of osteoblastic osteosarcoma (Sosa et al., 2022). These findings suggest that PSCs and bone marrow SSCs serve as the cell of origin for primary skeletal. The occurrence of primary skeletal tumors is predominantly observed in children and adolescents, coinciding with the active period of SSC activity. There is a possibility that SSCs are the cell of origin for primary skeletal tumors, with different SSCs serving as the cell of origin for distinct subsets of primary skeletal tumors.

## Conclusions and perspectives

With the identification of markers for SSCs, these pioneering studies have opened a window to understand SSCs, but many important issues remain unclear in this field: (i) the exact spaciotemporal features of SSCs require further research; (ii) the regulation of SSC fate by the niche remains unclear, including the interaction between SSCs and niche components such as endothelial cells, immune cells, and nerves; (iii) the role of SSCs in bone metastasis is still being explored. While it has been confirmed that Mfge8 secreted by a vSSC-lineage drives spinal metastasis in breast cancer, the role of SSCs in lung cancer and prostate cancer, which are also prone to bone metastasis, remains uncertain. Can small molecules targeting SSC-derived skeletal lineages be used to target cancer-driven bone metastasis in the future? (iv) It is still uncertain whether SSCs exhibit abnormalities in genetic bone diseases like osteogenesis imperfecta and Achondroplasia. To summarize, the research on SSCs has made noteworthy advancements in the last decade, shedding light on bone diseases and potentially leading to enhanced treatments.

## Abbreviations

αSMA: α-smooth muscle actin
*Acan*: Aggrecan
*Alpl*: alkaline phosphatase, biomineralization associated
BCSPs: bone, cartilage, and stromal progenitor cells
BMP2: bone morphogenetic protein-2
BMSCs: bone marrow stromal cells
CAR cells: Cxcl12-abundant reticular cells
CFU-F: colony-forming unit-fibroblast
CLPs: common lymphoid progenitors
*Col2a1*: type II collagen alpha 1 chain
*Ctsk*: cathepsin K
*Cxcl12*: C-X-C motif chemokine ligand 12
*DDR2*: discoidin domain-containing receptor 2
DeaLT: dual-recombinase-activated lineage tracing
EMB: embigin
eSSCs: embryonic skeletal stem cells
FACS: fluorescence-activated cell sorting
*Fgfr3*: fibroblast growth factor receptor 3
*FoxA2*: forkhead box A2
*Gli1*: GLI family zinc finger 1
HSCs: hematopoietic stem cells
IGF1: insulin-like growth factor-1
Ihh: Indian hedgehog
LKB1: liver kinase b1
Mfge8: protein milk fat globule EGF8
MSCs: mesenchymal stem cells
mTORC1: mechanistic target of rapamycin complex 1
OBs: osteoblasts
*Ocn*: osteocalcin
OCR: osteochondroreticular
ocSSCs: osteochondral SSCs
*Oln*: , osteolectin
*Osx*: oesterix
pre-BCSPs: precursor bone, cartilage, and stromal progenitor cells
pre-OBs: pre-osteoblasts
PSCs: periosteal stem cells
*PTHrP*: parathyroid hormone related peptide
*Ptpn11*: protein tyrosine phosphatase non-receptor type 11
pvSSCs: perivascular SSCs
*RanGAP1*: Ran GTPase activating protein 1
ScRNA-seq: single-cell RNA sequencing
SOC: secondary ossification center
*SOX9*: SRY-box transcription factor 9
*Sp7*: transcription factor 7
*Spp1*: secreted phosphoprotein 1
SSCs: skeletal stem cells
*STAT3*: signal transducer and activator of transcription 3
TNFα: tumor necrosis factor alpha
*Twist1*: twist family bHLH transcription factor 1
VEGFA: vascular endothelial growth factor A
vSSCs: vertebral SSCs
*Z24*: Zmpste24x

## References

[CIT0001] Akiyama H , Kim J-E, Nakashima K et al. Osteo-chondroprogenitor cells are derived from Sox9 expressing precursors. Proc Natl Acad Sci U S A 2005;102:14665–14670. 16203988 10.1073/pnas.0504750102 PMC1239942

[CIT0002] Allen MR , Hock JM, Burr DB. Periosteum: biology, regulation, and response to osteoporosis therapies. Bone 2004;35:1003–1012. 15542024 10.1016/j.bone.2004.07.014

[CIT0003] Ambrosi TH , Chan CKF. A seed-and-soil theory for blood ageing. Nat Cell Biol 2023;25:9–11. 36650380 10.1038/s41556-022-01062-z PMC10611483

[CIT0004] Ambrosi TH , Marecic O, McArdle A et al. Aged skeletal stem cells generate an inflammatory degenerative niche. Nature2021a;597:256–262. 34381212 10.1038/s41586-021-03795-7 PMC8721524

[CIT0005] Ambrosi TH , Sinha R, Steininger HM et al. Distinct skeletal stem cell types orchestrate long bone skeletogenesis. Elife2021b;10:e66063. 34280086 10.7554/eLife.66063 PMC8289409

[CIT0006] Armiento AR , Alini M, Stoddart MJ. Articular fibrocartilage—why does hyaline cartilage fail to repair? Adv Drug Deliv Rev 2019;146:289–305. 30605736 10.1016/j.addr.2018.12.015

[CIT0007] Arostegui M , Scott RW, Böse K et al. Cellular taxonomy of Hic1(+) mesenchymal progenitor derivatives in the limb: from embryo to adult. Nat Commun 2022;13:4989. 36008423 10.1038/s41467-022-32695-1 PMC9411605

[CIT0008] Baccin C , Al-Sabah J, Velten L et al. Combined single-cell and spatial transcriptomics reveal the molecular, cellular and spatial bone marrow niche organization. Nat Cell Biol 2020;22:38–48. 31871321 10.1038/s41556-019-0439-6 PMC7610809

[CIT0009] Bianco P. “Mesenchymal” stem cells. Annu Rev Cell Dev Biol 2014;30:677–704. 25150008 10.1146/annurev-cellbio-100913-013132

[CIT0010] Bianco P , Robey PG. Skeletal stem cells. Development 2015;142:1023–1027. 25758217 10.1242/dev.102210 PMC4360182

[CIT0011] Bok S , Yallowitz AR, Sun J et al. A multi-stem cell basis for craniosynostosis and calvarial mineralization. Nature 2023;621:804–812. 37730988 10.1038/s41586-023-06526-2 PMC10799660

[CIT0012] Chan CKF , Seo EY, Chen JY et al. Identification and specification of the mouse skeletal stem cell. Cell 2015;160:285–298. 25594184 10.1016/j.cell.2014.12.002 PMC4297645

[CIT0013] Chan CKF , Gulati GS, Sinha R et al. Identification of the human skeletal stem cell. Cell 2018;175:43–56.e21. 30241615 10.1016/j.cell.2018.07.029 PMC6400492

[CIT0014] Clarke B. Normal bone anatomy and physiology. Clin J Am Soc Nephrol 2008;3:S131–S139. 18988698 10.2215/CJN.04151206 PMC3152283

[CIT0015] Compston JE , McClung MR, Leslie WD. Osteoporosis. Lancet (London, England) 2019;393:364–376. 30696576 10.1016/S0140-6736(18)32112-3

[CIT0016] Debnath S , Yallowitz AR, McCormick J et al. Discovery of a periosteal stem cell mediating intramembranous bone formation. Nature 2018;562:133–139. 30250253 10.1038/s41586-018-0554-8 PMC6193396

[CIT0017] Eggenhofer E , Luk F, Dahlke MH et al. The life and fate of mesenchymal stem cells. Front Immunol 2014;5:148. 24904568 10.3389/fimmu.2014.00148 PMC4032901

[CIT0018] Feil S , Valtcheva N, Feil R. Inducible Cre mice. Methods Mol Biol 2009;530:343–363. 19266339 10.1007/978-1-59745-471-1_18

[CIT0019] Gerber HP , Vu TH, Ryan AM et al. VEGF couples hypertrophic cartilage remodeling, ossification and angiogenesis during endochondral bone formation. Nat Med 1999;5:623–628. 10371499 10.1038/9467

[CIT0020] Greenblatt MB , Ono N, Ayturk UM et al. The unmixing problem: a guide to applying single-cell RNA sequencing to bone. J Bone Miner Res 2019;34:1207–1219. 31336008 10.1002/jbmr.3802 PMC6658136

[CIT0021] Gulati GS , Murphy MP, Marecic O et al. Isolation and functional assessment of mouse skeletal stem cell lineage. Nat Protoc 2018;13:1294–1309. 29748647 10.1038/nprot.2018.041 PMC6530903

[CIT0022] Han Y , Feng H, Sun J et al. Lkb1 deletion in periosteal mesenchymal progenitors induces osteogenic tumors through mTORC1 activation. J Clin Invest 2019;129:1895–1909. 30830877 10.1172/JCI124590 PMC6486357

[CIT0023] He X , Bougioukli S, Ortega B et al. Sox9 positive periosteal cells in fracture repair of the adult mammalian long bone. Bone 2017;103:12–19. 28627474 10.1016/j.bone.2017.06.008 PMC6435293

[CIT0024] He L , Li Y, Huang X et al. Genetic lineage tracing of resident stem cells by DeaLT. Nat Protoc 2018;13:2217–2246. 30250288 10.1038/s41596-018-0034-5

[CIT0025] He J , Yan J, Wang J et al. Dissecting human embryonic skeletal stem cell ontogeny by single-cell transcriptomic and functional analyses. Cell Res 2021;31:742–757. 33473154 10.1038/s41422-021-00467-z PMC8249634

[CIT0026] Jacome-Galarza CE , Percin GI, Muller JT et al. Developmental origin, functional maintenance and genetic rescue of osteoclasts. Nature 2019;568:541–545. 30971820 10.1038/s41586-019-1105-7 PMC6910203

[CIT0027] Jeffery EC , Mann TLA, Pool JA et al. Bone marrow and periosteal skeletal stem/progenitor cells make distinct contributions to bone maintenance and repair. Cell Stem Cell 2022;29:1547–1561.e6. 36272401 10.1016/j.stem.2022.10.002

[CIT0028] Jin A , Xu H, Gao X et al. ScRNA-Seq reveals a distinct osteogenic progenitor of alveolar bone. J Dent Res 2023;102:645–655. 37148259 10.1177/00220345231159821

[CIT0029] Jing D , Chen Z, Men Y et al. Response of Gli1(+) suture stem cells to mechanical force upon suture expansion. J bone Miner Res Off J Am Soc Bone Miner Res 2022;37:1307–1320. 10.1002/jbmr.4561 35443291

[CIT0030] Jones DC , Wein MN, Glimcher LH. Schnurri-3 is an essential regulator of osteoblast function and adult bone mass. Ann Rheum Dis 2007;66:iii49–iii51. 17934096 10.1136/ard.2007.078352 PMC2095286

[CIT0031] Josephson AM , Bradaschia-Correa V, Lee S et al. Age-related inflammation triggers skeletal stem/progenitor cell dysfunction. Proc Natl Acad Sci U S A 2019;116:6995–7004. 30894483 10.1073/pnas.1810692116 PMC6452701

[CIT0032] Kara N , Xue Y, Zhao Z et al. Endothelial and Leptin Receptor(+) cells promote the maintenance of stem cells and hematopoiesis in early postnatal murine bone marrow. Dev Cell 2023;58:348–360.e6. 36868235 10.1016/j.devcel.2023.02.003 PMC10035381

[CIT0033] Khosla S , Farr JN, Tchkonia T et al. The role of cellular senescence in ageing and endocrine disease. Nat Rev Endocrinol 2020;16:263–275. 32161396 10.1038/s41574-020-0335-y PMC7227781

[CIT0034] Kretzschmar K , Watt FM. Lineage tracing. Cell 2012;148:33–45. 22265400 10.1016/j.cell.2012.01.002

[CIT0035] Li X , Yang S, Yuan G et al. Type II collagen-positive progenitors are important stem cells in controlling skeletal development and vascular formation. Bone Res 2022;10:46. 35739091 10.1038/s41413-022-00214-z PMC9226163

[CIT0036] Liu H , Li P, Zhang S et al. Prrx1 marks stem cells for bone, white adipose tissue and dermis in adult mice. Nat Genet 2022;54:1946–1958. 36456880 10.1038/s41588-022-01227-4

[CIT0037] Logan M , Martin JF, Nagy A et al. Expression of Cre Recombinase in the developing mouse limb bud driven by a Prxl enhancer. Genesis 2002;33:77–80. 12112875 10.1002/gene.10092

[CIT0038] Ma L , Chang Q, Pei F et al. Skull progenitor cell-driven meningeal lymphatic restoration improves neurocognitive functions in craniosynostosis. Cell Stem Cell 2023;30:1472–1485.e7. 37863055 10.1016/j.stem.2023.09.012 PMC10842404

[CIT0039] Maes C , Kobayashi T, Selig MK et al. Osteoblast precursors, but not mature osteoblasts, move into developing and fractured bones along with invading blood vessels. Dev Cell 2010;19:329–344. 20708594 10.1016/j.devcel.2010.07.010 PMC3540406

[CIT0040] Maruyama T. Stem cells of the suture mesenchyme in craniofacial bone development, repair and regeneration. Keio J Med 2019;68:42. 31243185 10.2302/kjm.68-003-ABST

[CIT0041] Maruyama T , Stevens R, Boka A et al. BMPR1A maintains skeletal stem cell properties in craniofacial development and craniosynostosis. Sci Transl Med 2021;13:583. 10.1126/scitranslmed.abb4416 PMC8590202 33658353

[CIT0042] Matsushita Y , Chu AKY, Tsutsumi-Arai C et al. The fate of early perichondrial cells in developing bones. Nat Commun 2022;13:7319. 36443296 10.1038/s41467-022-34804-6 PMC9705540

[CIT0043] Matsushita Y , Liu J, Chu AKY et al. Bone marrow endosteal stem cells dictate active osteogenesis and aggressive tumorigenesis. Nat Commun 2023;14:2383. 37185464 10.1038/s41467-023-38034-2 PMC10130060

[CIT0044] Matthews BG , Novak S, Sbrana FV et al. Heterogeneity of murine periosteum progenitors involved in fracture healing. Elife 2021;10:e58534. 33560227 10.7554/eLife.58534 PMC7906599

[CIT0045] McLellan MA , Rosenthal NA, Pinto AR. Cre-loxP-mediated recombination: general principles and experimental considerations. Curr Protoc Mouse Biol 2017;7:1–12. 28252198 10.1002/cpmo.22

[CIT0046] McLeod CM , Mauck RL. On the origin and impact of mesenchymal stem cell heterogeneity: new insights and emerging tools for single cell analysis. Eur Cell Mater 2017;34:217–231. 29076514 10.22203/eCM.v034a14 PMC7735381

[CIT0047] Men Y , Wang Y, Yi Y et al. Gli1+ periodontium stem cells are regulated by osteocytes and occlusal force. Dev Cell 2020;54:639–654.e6. 32652075 10.1016/j.devcel.2020.06.006

[CIT0048] Méndez-Ferrer S , Michurina TV, Ferraro F et al. Mesenchymal and haematopoietic stem cells form a unique bone marrow niche. Nature 2010;466:829–834. 20703299 10.1038/nature09262 PMC3146551

[CIT0049] Mitchell CA , Verovskaya EV, Calero-Nieto FJ et al. Stromal niche inflammation mediated by IL-1 signalling is a targetable driver of haematopoietic ageing. Nat Cell Biol 2023;25:30–41. 36650381 10.1038/s41556-022-01053-0 PMC7614279

[CIT0050] Mizoguchi T , Pinho S, Ahmed J et al. Osterix marks distinct waves of primitive and definitive stromal progenitors during bone marrow development. Dev Cell 2014;29:340–349. 24823377 10.1016/j.devcel.2014.03.013 PMC4051418

[CIT0051] Mizuhashi K , Ono W, Matsushita Y et al. Resting zone of the growth plate houses a unique class of skeletal stem cells. Nature 2018;563:254–258. 30401834 10.1038/s41586-018-0662-5 PMC6251707

[CIT0052] Mo C , Guo J, Qin J et al. Single-cell transcriptomics of LepR-positive skeletal cells reveals heterogeneous stress-dependent stem and progenitor pools. EMBO J 2022;41:e108415. 34957577 10.15252/embj.2021108415 PMC8844986

[CIT0053] Murphy MP , Koepke LS, Lopez MT et al. Articular cartilage regeneration by activated skeletal stem cells. Nat Med 2020;26:1583–1592. 32807933 10.1038/s41591-020-1013-2 PMC7704061

[CIT0054] Newton PT , Li L, Zhou B et al. A radical switch in clonality reveals a stem cell niche in the epiphyseal growth plate. Nature 2019;567:234–238. 30814736 10.1038/s41586-019-0989-6

[CIT0055] Ng LJ , Wheatley S, Muscat GE et al. SOX9 binds DNA, activates transcription, and coexpresses with type II collagen during chondrogenesis in the mouse. Dev Biol 1997;183:108–121. 9119111 10.1006/dbio.1996.8487

[CIT0056] Ono N , Ono W, Nagasawa T et al. A subset of chondrogenic cells provides early mesenchymal progenitors in growing bones. Nat Cell Biol 2014;16:1157–1167. 25419849 10.1038/ncb3067 PMC4250334

[CIT0057] Pineault KM , Song JY, Kozloff KM et al. Hox11 expressing regional skeletal stem cells are progenitors for osteoblasts, chondrocytes and adipocytes throughout life. Nat Commun 2019;10:3168. 31320650 10.1038/s41467-019-11100-4 PMC6639390

[CIT0058] Pittenger MF , Mackay AM, Beck SC et al. Multilineage potential of adult human mesenchymal stem cells. Science 1999;284:143–147. 10102814 10.1126/science.284.5411.143

[CIT0059] Shen B , Tasdogan A, Ubellacker JM et al. A mechanosensitive peri-arteriolar niche for osteogenesis and lymphopoiesis. Nature 2021;591:438–444. 33627868 10.1038/s41586-021-03298-5 PMC7979521

[CIT0060] Shi Y , He G, Lee W-C et al. Gli1 identifies osteogenic progenitors for bone formation and fracture repair. Nat Commun 2017;8:2043. 29230039 10.1038/s41467-017-02171-2 PMC5725597

[CIT0061] Shu HS , Liu YL, Tang XT et al. Tracing the skeletal progenitor transition during postnatal bone formation. Cell Stem Cell 2021;28:2122–2136.e3. 34499868 10.1016/j.stem.2021.08.010

[CIT0062] Soriano P. Generalized lacZ expression with the ROSA26 Cre reporter strain. Nat Genet 1999;21:70–71. 9916792 10.1038/5007

[CIT0063] Sosa BR , Wang Z, Healey JH et al. A subset of osteosarcoma bears markers of CXCL12-abundant reticular cells. JBMR Plus 2022;6:e10596. 35309866 10.1002/jbm4.10596 PMC8914147

[CIT0064] Sun J , Hu L, Bok S et al. A vertebral skeletal stem cell lineage driving metastasis. Nature 2023;621:602–609. 37704733 10.1038/s41586-023-06519-1 PMC10829697

[CIT0065] Takahashi A , Nagata M, Gupta A et al. Autocrine regulation of mesenchymal progenitor cell fates orchestrates tooth eruption. Proc Natl Acad Sci U S A 2019;116:575–580. 30509999 10.1073/pnas.1810200115 PMC6329940

[CIT0066] Tevlin R , Seo EY, Marecic O et al. Pharmacological rescue of diabetic skeletal stem cell niches. Sci Transl Med 2017;9:eaag2809. 28077677 10.1126/scitranslmed.aag2809 PMC5725192

[CIT0067] Tikhonova AN , Dolgalev I, Hu H et al. The bone marrow microenvironment at single-cell resolution. Nature 2019;569:222–228. 30971824 10.1038/s41586-019-1104-8 PMC6607432

[CIT0068] Tsukasaki M , Komatsu N, Negishi-Koga T et al. Periosteal stem cells control growth plate stem cells during postnatal skeletal growth. Nat Commun 2022;13:4166. 35851381 10.1038/s41467-022-31592-x PMC9293991

[CIT0069] Wang K , Xu C, Xie X et al. Axin2+ PDL cells directly contribute to new alveolar bone formation in response to orthodontic tension force. J Dent Res 2022;101:695–703. 35001706 10.1177/00220345211062585 PMC9124907

[CIT0070] Wilk K , Yeh S-CA, Mortensen LJ et al. Postnatal calvarial skeletal stem cells expressing PRX1 reside exclusively in the calvarial sutures and are required for bone regeneration. Stem Cell Rep 2017;8:933–946. 10.1016/j.stemcr.2017.03.002 PMC5390237 28366454

[CIT0071] Worthley DL , Churchill M, Compton JT et al. Gremlin 1 identifies a skeletal stem cell with bone, cartilage, and reticular stromal potential. Cell 2015;160:269–284. 25594183 10.1016/j.cell.2014.11.042 PMC4436082

[CIT0072] Xie X , Xu C, Zhao L et al. Axin2-expressing cells in the periodontal ligament are regulated by bone morphogenetic protein signalling and play a pivotal role in periodontium development. J Clin Periodontol 2022;49:945–956. 35634660 10.1111/jcpe.13666

[CIT0073] Yahara Y , Barrientos T, Tang YJ et al. Erythromyeloid progenitors give rise to a population of osteoclasts that contribute to bone homeostasis and repair. Nat Cell Biol 2020;22:49–59. 31907410 10.1038/s41556-019-0437-8 PMC6953622

[CIT0074] Yang W , Wang J, Moore DC et al. Ptpn11 deletion in a novel progenitor causes metachondromatosis by inducing hedgehog signalling. Nature 2013;499:491–495. 23863940 10.1038/nature12396 PMC4148013

[CIT0075] Yang L , Tsang KY, Tang HC et al. Hypertrophic chondrocytes can become osteoblasts and osteocytes in endochondral bone formation. Proc Natl Acad Sci U S A 2014;111:12097–12102. 25092332 10.1073/pnas.1302703111 PMC4143064

[CIT0076] Yu M , Ma L, Yuan Y et al. Cranial suture regeneration mitigates skull and neurocognitive defects in craniosynostosis. Cell 2021;184:243–256.e18. 33417861 10.1016/j.cell.2020.11.037 PMC7891303

[CIT0077] Yue R , Zhou BO, Shimada IS et al. Leptin receptor promotes adipogenesis and reduces osteogenesis by regulating mesenchymal stromal cells in adult bone marrow. Cell Stem Cell 2016;18:782–796. 27053299 10.1016/j.stem.2016.02.015

[CIT0078] Zeller R , López-Ríos J, Zuniga A. Vertebrate limb bud development: moving towards integrative analysis of organogenesis. Nat Rev Genet 2009;10:845–858. 19920852 10.1038/nrg2681

[CIT0079] Zhao Q , Eberspaecher H, Lefebvre V et al. Parallel expression of Sox9 and Col2a1 in cells undergoing chondrogenesis. Dev Dyn 1997;209:377–386. 9264261 10.1002/(SICI)1097-0177(199708)209:4<377::AID-AJA5>3.0.CO;2-F

[CIT0080] Zhao H , Feng J, Ho T-V et al. The suture provides a niche for mesenchymal stem cells of craniofacial bones. Nat Cell Biol 2015;17:386–396. 25799059 10.1038/ncb3139 PMC4380556

[CIT0081] Zhou BO , Yue R, Murphy MM et al. Leptin-receptor-expressing mesenchymal stromal cells represent the main source of bone formed by adult bone marrow. Cell Stem Cell2014a;15:154–168. 24953181 10.1016/j.stem.2014.06.008 PMC4127103

[CIT0082] Zhou X , von der Mark K, Henry S et al. Chondrocytes transdifferentiate into osteoblasts in endochondral bone during development, postnatal growth and fracture healing in mice. PLoS Genet2014b;10:e1004820. 25474590 10.1371/journal.pgen.1004820 PMC4256265

[CIT0083] Zhou S , Dai Q, Huang X et al. STAT3 is critical for skeletal development and bone homeostasis by regulating osteogenesis. Nat Commun 2021;12:6891. 34824272 10.1038/s41467-021-27273-w PMC8616950

